# Measuring management practices in primary health care facilities – development and validation of management practices scorecard in Nigeria

**DOI:** 10.1080/16549716.2020.1763078

**Published:** 2020-06-08

**Authors:** Shunsuke Mabuchi, Olakunle Alonge, Yusuke Tsugawa, Sara Bennett

**Affiliations:** a Global Delivery Programs, Bill and Melinda Gates Foundation, Seattle, WA, USA; b International Health, Johns Hopkins Bloomberg School of Public Health, Baltimore, MD, USA; c Medicine, UCLA, Los Angeles, CA, USA

**Keywords:** Primary health care facilities, performance-based financing, health facility management, scorecard, factor analysis, Nigeria

## Abstract

**Background:**

In low- and middle-income countries, there is scarcity of validated and reliable measurement tools for health facility management, and many interventions to improve primary health care (PHC) facilities are designed without adequate evidence base on what management practices are critical.

**Objective:**

This article developed and validated a scorecard to measure management practices at primary health care facilities under the performance-based financing (PBF) scheme in Nigeria.

**Methods:**

Relevant management practice domains and indicators for PHC facilities were determined based on literature review and a prior qualitative study conducted in Nigeria. The domains and indicators were tested for face validity via experts review and organized into an interviewer-administered scorecard. A stratified random sampling of PHC facilities in three States in Nigeria was conducted to assess the reliability and construct validity of the scorecard. Inter-rater reliability using inter-class correlation (ICC) (1, k) was assessed with one-way ANOVA. Exploratory factor analysis (EFA) was conducted to assess the construct validity, and an updated factor structure were developed.

**Results:**

32 indicators and 6 management practice domains were initially described. Ordinal responses were derived for each indicator. Data on the scorecard were obtained from 111 PHC facilities. The ICC of mean ratings for each team of judges was 0.94. The EFA identified 6 domains (Stakeholder engagement and communication; Community-level activities; Update of plan and target; Performance management; Staff attention to planning, target, and performance; and Drugs and financial management) and reduced the number of indicators to 17. The average communality of selected items was 0.45, and item per factor ratio was 17:6.

**Conclusions:**

Despite a few areas for further refinement, this paper presents a reliable and valid scorecard for measuring management practices in PHC facilities. The scorecard can be applied for routine supervisory visits to PHC facilities, and can help accumulate knowledge on facility management, how it affects performance, and how it may be strengthened.

## Background

In low- and middle-income countries (LMICs), numerous initiatives have been introduced with the aim of improving the quality and performance of primary health services. However, determinants of primary health facility performance in resource-limited settings have not been well understood [1]. Among many factors that potentially determine the performance of primary health facilities, one of the most under-researched areas is health facility management. Several studies [1–5] have identified specific health facility management practices that are associated with the improvement of health facility performance including: (i) engaging and problem-solving with local stakeholders [3], particularly with community leaders [5]; (ii) building a system of accountability [4], through performance management activities [5]; (iii) motivating health workers for change [2,3,5]; (iv) building work around teams and creating a sense of belonging, trust and respect [1,3,5]; (v) providing management support [1,3,5]; and (vi) improving health facility managers leadership competency to build a supportive environment for staff [2,4,5].

Much of the research on primary health care (PHC) management practices in low and middle-income countries (LMICs) is qualitative in nature, employing case studies and realist evaluations to understand which types of management practices work in which settings [1–5]. Quantitative empirical studies aimed at measuring these critical health facility management practices and exploring the relationship between such practices and health facility performance are limited primarily to inpatient settings in developed countries [6–17]. Without a validated and reliable measurement tool, many interventions to improve the management and performance of primary health care facilities in LMICs are designed without an adequate evidence base on what management practices are critical for improving health facility performance. The lack of measurement tool for management practices further limits the assessment of health system strengthening (HSS) interventions in LMICs.

Performance-based financing (PBF) is an example of such HSS interventions and has increasingly been used as a major approach for improving health facility performance in LMICs. It is defined as “fee-for-service conditional on quality of care’ [18]. In Nigeria, PBF was rolled out in 3 out of 36 States (Adamawa, Nasarawa, and Ondo States) to improve the quality and quantity of primary health care centers (PHCCs) services in 2014. The major design features of the PBF scheme in Nigeria include: (i) the provision of finance to health facilities on top of existing budgets based on the quantity and quality of services provided; and (ii) increased autonomy for PHCCs so that they may use the received funds to improve health services. Part of the funding received can also be allocated to motivate health workers based on their performance and responsibilities [19]. Given that PBF provides resources and autonomy for PHCCs to manage resources, management practices at the PHCCs covered by such PBF schemes will be even more important than facilities without such a platform. How PBF works at the health facility level and how health facility management influences performance under PBF have been regarded as a ‘Black Box’ [20]. A reliable measurement tool is required to unveil the dynamics between PBF, health center management, and performance.

The objective of our study was to develop and validate a scorecard to measure management practices at primary health care facilities under the PBF scheme in Nigeria. While the scorecard was designed to include a few measures of PBF-specific activities (such as the development of business plan and use of PBF funds), broader measures of management practices applicable to diverse PHCC programs were also included. We hope that the scorecard will be useful for the assessment of management practices at PHCCs, and improve our understanding of how PBF and similar HSS interventions work to influence health facility management in LMICs.

## Methods

This study was conducted in 2014–2016. The management practices scorecard was developed through a two-step process. In the first step, we conducted a literature review to: (i) examine existing tools; and (ii) develop management practices areas and sub-areas that could be built on to develop a management practices scorecard for PBF in Nigeria. The authors reviewed literature collected through a PubMed and Google scholar search for the years 1996–2016. The search combined terms related to: (i) measurement tools; (ii) facility management practice and management competencies; and (iii) primary health care. We applied this search to literature from both developed and developing countries. We selected a tool to form the foundation for the scorecard based on the following criteria: (i) the tool had gone through a validation process that linked management measures to health facility performance; and (ii) the indicators and measurement approach were consistent with findings from a qualitative case study at PHCCs in Nigeria [5] that identified community engagement, performance management and staff management as key management practices. Further, based on over 40 relevant publications in peer-reviewed journals on management competencies, we developed a list of important management practice areas for PHCCs. We also reviewed the PBF literature to identify additional management practice areas for managing PBF schemes in health facilities. We extracted all the elements of management practices mentioned in the relevant publications, and organized them into a shortlist of management practice areas and sub-areas under each area. We also selected relevant indicators under each sub-area based on the review of existing tools.

As the second step, we developed additional indicators, scoring criteria using ordinal responses, and a scoring grid based on the key findings of the qualitative study [5]. For example, the case study research found that high-performing PHCCs carry out frequent outreach activities, visit targeted households in each outreach, change services based on the feedback from patients, and carry out many strategic activities to recruit and retain patients such as creating incentives for women to deliver at PHCCs, individualized follow-up of pregnant women, year-end celebrations. These practices were converted to indicators to measure community/client engagement, and ordinal responses for each indicator were developed by comparing relevant practices between high versus low-performing PHCCs observed in the qualitative study.

We tested the face validity of the scorecard through a review of health system personnel in Nigeria including staff of the National Primary Health Care Development Agency (NPHCDA), and State Primary Health Care Development Agencies (SPHCDAs) in Adamawa, Nasarawa, and Ondo states of Nigeria; PBF technical assistance consultants; the World Bank’s Nigeria health team; and health systems experts at the Johns Hopkins University. We then divided the scorecard into two separate questionnaires to be administered (i) to officers in charge (OICs) and (ii) non-OIC health workers. We pilot-tested the questionnaires in six PHCCs under PBF in Nasarawa state that were not included in the main phase of data collection and revised the questionnaires for clarity and interpretation based on feedback from the pilot-test. The revised questionnaires, evaluation criteria and scoring grid were scripted into electronic templates on hand-held devices to facilitate data collection and reduce errors in scoring and aggregation of the scores.

### Sampling and data collection

We proposed a sample size (N) of 111 PHCCs to explore a scorecard with 32 indicators or questions (p) for a N:p ratio of 3 as described by Arrindell and van der Ende [21] to be adequate for demonstrating the validity and reliability of questionnaires designed for identifying latent constructs with alpha level of 0.05. We selected the 111 PHCCs from 457 PHCCs that have been implementing PBF in the three Nigerian States (Adamawa, Nasarawa, and Ondo). We used a stratified random sampling technique to allocate the 111 PHCCs (Adamawa: 54, Nasarawa: 21 and Ondo: 36) based on the number of PHCCs under the PBF scheme in each State.

In the EFA presented below, average communality of selected items was 0.45. The items per factor ratio (p:r ratio) in the model presented in Table 4 is 17:6. For EFA with low communality case (less than 0.4), an empirical study suggested that N = 100 and N = 200 are needed to have over 95% convergences for 20:3 and 10:3 p:r ratios respectively [22]. They also found that for p:r ratio 10:3 or 20:3, a sample size (N = 60) could still result in over 99% convergence if the level of commonality is wide or high. As the commonality of this EFA result is higher than 0.4, 17:6 p:r ratio may require a little over 100, which is consistent with the sample size of our study, N = 111.

**Table 1.** Synthesized key elements of critical primary health facility management.

Key elements	Synthesized Definition	Reference
Problem solving	Analyze issues and make decisions systematically using evidence, encourage staff and achieve results.	Baldridge performance excellence program, 2011; Management Sciences for health, 1998; Karsten, 2010; McCarthy et al, 2009; Office for Health Management, 2004; Omoike et al, 2011; Schmalenberg, 2009; Sherman et al, 2007; Zori et al, 2010
Communication	Communicate facility’s vision, values and key decisions and influence health workers, while engaging in frank, two-way communication throughout the facility.	Baldridge performance excellence program, 2011; Kramer et al, 2007; McCarthy et al, 2009; Office for Health Management, 2004; Omoike et al, 2011; Pillay, 2010; Sherman et al, 2007; Squires, 2010; Zori et al, 2010.
Staff and team management	Create opportunities for learning, motivate and coach health workers and promote cohesion and team work. Assign appropriate roles and responsibilities.	Baldridge performance excellence program, 2011; Management Sciences for health, 1998; Karsten, 2010; McCarthy et al, 2009; Office for Health Management, 2004; Omoike et al, 2011; Schmalenberg, 2009; Sherman et al, 2007; Squires, 2010.
Planning	Set clear target, and plan resources efficiently and effectively within a specified time frame. Co-ordinate and schedule activities.	Baldridge performance excellence program, 2011; Karsten, 2010; McCarthy et al, 2009; NHS Institute for Innovation and Improvement and Academy of Medical Royal Colleges,2010; Office for Health Management, 2004; Omoike et al, 2011; Pillay, 2010; Squires, 2010.
Performance management	Measure performance, conduct formal performance reviews, mobilize resources and lead on proactive improvements.	Baldridge performance excellence program, 2011; Management Sciences for health, 1998; NHS Institute for Innovation and Improvement and Academy of Medical Royal Colleges,2010; Omoike et al, 2011; Pillay, 2010; Squires, 2010.
Relationship building and resource mobilization	Develop and manage networks and relationships. Can mobilize necessary resources such as HR, equipment and supplies when necessary.	McCarthy et al, 2009; NHS Institute for Innovation and Improvement and Academy of Medical Royal Colleges,2010; Office for Health Management, 2004; Schmalenberg, 2009
Financial management	Record, manage and balance revenue and expense to enable continuous improvement.	McCarthy et al, 2009; Office for Health Management, 2004; Schmalenberg, 2009

**Table 2.** Areas and indicators of the developed management practices scorecard.

Areas	Sub-Areas	ID	Item	Mean	sd
1. Community/Client Engagement	1.1. Community outreach	S1	Outreach	1.77	0.55
		S2	Household visit	2.30	0.80
	1.2. Community trust and satisfaction	S3	Listening and responding to client feedback	2.21	0.63
	1.3. Client recruitment/retention	S4	Patient recruitment and retention activities	1.56	0.57
2. Stakeholder Engagement	2.1. Engagement with Community Leaders	S5	Meetings with community leaders	1.94	0.83
		S6	Request to community leaders	1.88	0.74
		S7	Activities to encourage support from community leaders	2.05	0.54
	2.2. Supervisor engagement	S8	Meetings with supervisors	1.94	0.74
		S9	Request to supervisors	1.54	0.72
3. Staff management	3.1. Management of staff and working environment	S10	Staff involvement in bonus decision	2.72	0.51
		S11	Team work building	1.91	0.35
		S12	Efforts to improve staff working environment	2.07	0.35
	3.2. Staff communication	S13	Feedback to OIC	2.40	0.66
		S14	Responses from OIC to feedback	2.55	0.52
		S15	Open communication	2.77	0.44
	3.3. Recognition, Rewarding and punishment of staff	S16	PBF bonus allocation	2.68	0.70
		S17	Rewarding of high-performing staff	1.90	0.71
		S18	Addressing low-performing staff	1.83	0.54
4. Planning and Target Setting	4.1. Planning	S19	Business plan update	2.77	0.43
		S20	Business plan content	2.77	0.46
		S21	Staff attention to business plan	1.60	0.59
	4.2. Target setting	S22	Target update	2.61	0.49
		S23	Setting stretch/achievable targets	2.07	0.46
		S24	Staff attention to targets	1.50	0.60
5. Performance Management	5.1. Performance tracking	S25	Visualization of performance data	2.26	0.55
		S26	Staff attention to performance	1.69	0.77
	5.2. Performance review	S27	Regular performance review meeting	2.57	0.64
		S28	Performance review discussions	2.18	0.73
6. Use of funds and financial management	6.1. Use of funds	S29	Drug management	2.49	0.70
		S30	Use of PBF funds to attract patient and build trust	2.02	0.33
	6.2. Financial Management	S31	Financial record update	2.34	0.77
		S32	Financial record content	2.24	0.81

sd: standard deviation of the responses from 222 respondents from 111 facilities.

**Table 3.** Result of exploratory factor analysis for six factors with PROMAX rotation.

			EFA Factor Loadings					
Latent Factors	No.	Items	A	B	C	D	E	F
A. Stakeholder engagement and communication	S5	Meetings with community leaders	**0.49**	0.30	−0.11	−0.14	0.18	0.01
	S6	Request to community leaders	**0.45**	0.03	0.11	0.16	−0.02	−0.13
	S8	Meetings with supervisors	**0.44**	0.08	−0.01	−0.02	0.02	−0.27
	S14	Responses from OIC to staff feedback	**0.52**	−0.09	−0.13	0.11	0.03	0.14
B. Community-level activities	S7	Activities to encourage support from community leaders	0.22	**0.59**	−0.05	−0.07	−0.11	0.05
	S30	Use of PBF funds to attract patient and build trust	−0.13	**0.55**	0.08	0.25	0.01	0.13
C. Update of plan and target	S19	Business plan update	0.15	0.11	**0.62**	0.11	−0.03	0.11
	S22	Target update	0.02	0.02	**1.03**	−0.15	0.08	−0.12
D. Performance management	S23	Setting stretch/achievable targets	0.07	−0.13	0.21	**0.41**	0.03	0.12
	S28	Performance review discussions	0.24	0.06	0.01	**0.50**	0.16	0.02
	S18	Addressing low-performing staff	0.05	0.25	0.07	**0.45**	−0.22	−0.01
E. Staff attention to plan, target, and performance	S21	Staff attention to business plan	0.07	0.06	0.11	0.03	**0.56**	−0.01
	S24	Staff attention to targets	0.04	0.01	0.02	−0.05	**0.62**	−0.05
	S26	Staff attention to performance	0.00	−0.07	−0.04	0.09	**0.67**	0.13
F. Drugs and financial management	S29	Drug management	0.15	0.09	0.02	0.02	0.06	**0.42**
	S31	Financial record update	−0.19	0.00	−0.00	0.01	−0.03	**0.86**
	S32	Financial record content	−0.11	−0.00	0.02	0.06	0.03	**0.82**

**Table 4.** Comparison of factor and indicator structure from EFA with original scorecard.

Latent Factors	No.	Indicators	Analysis/Comparison with Original Scorecard
A. Stakeholder engagement and communication (Factor 2)	S5	Meetings with community leaders	S5, S6, and S8 relate to external stakeholder engagement (e.g. community, community leader, local government supervisor), while S14 relates to engagement with internal PHCC staff (responsiveness and rewarding to staff). Unlike the structure in the original scorecard that treats them separately, they seem to have a common latent factor on engagement and communication with external and internal stakeholders.
	S6	Request to community leaders	
	S8	Meetings with supervisors	
	S14	Responses from OIC to staff feedback	
B. Community-level activities (Factor 5)	S7	Activities to encourage support from community leaders	S7 and S30 have commonalities as their scoring criteria refer to community – level activities, such as incentives to community leaders (S7) and use of funds to attract patients to facilities and gain trust from community (S30).
	S30	Use of PBF funds to attract patient and build trust	
C. Update of plan and target (Factor 3)	S19	Business plan update	S19 (Business plan update) and S22 (Target update) show clear grouping related to frequent update of plan and target.
	S22	Target update	
D. Performance management (Factor 6)	S23	Setting stretch/achievable targets	S18 (Addressing low-performing staff), S23 (setting stretch/achievable targets), and S28 (Performance review discussions) are related to performance management, which is consistent with the original factor structure.
	S28	Performance review discussions	
	S18	Addressing low-performing staff	
E. Staff attention to plan, target, and performance (Factor 4)	S21	Staff attention to business plan	S21, S24, and S26 are all related to staff attention to plan, target, and performance of the PHCCs. This is different from the way the original scorecard was structured, but suggests importance of communication to and involvement of staff in planning, target setting, and performance review to raise their attention.
	S24	Staff attention to targets	
	S26	Staff attention to performance	
F. Drugs and financial management (Factor 1)	S29	Drug management	A group of S29, S31, S32 is consistent with the original scorecard, related to how PHCCs manage drugs and funds available for them.
	S31	Financial record update	
	S32	Financial record content	

Twelve interviewers were recruited to form 6 survey teams of 2 interviewers. The interviewers were trained for five days, including two days of field-testing of the questionnaires. Data collection was conducted during April and May 2016. The questionnaires were administered to the OIC of a selected PHCC, and a randomly selected non-OIC health worker who had worked for the PHCC for more than one year. The non-OIC health worker interviews were carried out in an isolated room or outside the PHCC without the presence of other staff. After having administered each questionnaire, the two interviewers scored the PHCC using the scoring grid and criteria separately without consultation with each other – individual scores were used for assessing inter-rater agreement. Thereafter, the two interviewers discussed the scores for each question and agreed on the final consolidated scores. Interviewers took short notes of responses to all questions, and the final scores were reviewed and validated by the lead author and a contracted data collection firm by referring to the short notes.

The original management practices measurement tool (from which indicators were derived for this scorecard) assumed interviews would be conducted by individuals with education and work experience in healthcare, such as masters or doctoral degrees in public health, medicine, or business administration to increase the reliability of the score [8]. Given that such resources are not easily available in developing countries, revisions were made to the scorecard so that: (i) scoring criteria for open-ended questions were defined clearly to reduce potential inter-rater reliability issues; and (ii) in addition to questions for OICs, we developed questions for non-OIC health workers to assess practices by OICs and reduce social desirability bias.

### Data analysis

We assessed descriptive statistics (arithmetic mean and standard deviation) for all indicators included in the scorecard for the 111 PHCCs. Thereafter, we assessed the inter-rater reliability of the scorecard using inter-class correlation (ICC) (1, k) for two judges and not the same judges for all PHCCs, with one-way ANOVA. The range, mean, standard deviation and histograms of the scores for each indicator were also examined to understand the distribution of the scores.

We then carried out exploratory factor analyses (EFA) to assess the construct validity of the scorecard. We estimated principal components for the indicators in the scorecard and selected the number of factors to retain based on eigenvalues (greater than 1) and a scree plot. A factor analysis with Iterated Principal Factors (IPF) method and PROMAX rotation was used to redefine the factors to improve their interpretability. We reviewed factor loadings to look into groups of indicators loaded on the same latent factors and to drop items with low loading (loading < 0.40) [23]. Also, cross-loading items with values ≥ 0.32 on at least two factors were deleted, especially if there were other items with factor loadings of 0.50 or greater [24]. The result of the EFA was used to develop an updated factor structure with a smaller number of items. We compared the updated factor structure with the original management practices scorecard we developed, and literature review results, to examine whether the updated structure can measure important management practices comprehensively. All analyses were conducted using STATA version 14 (Stata Corp).

## Results

### Scorecard development

#### Step 1: Literature review for tool selection and identification of management practices areas

The number of tools to measure management practices of health facilities was very limited. We found three instruments for health facilities in developed countries that had been validated, whose results were published in peer-reviewed journals – (i) the Management Practices Measurement tool [6–8]; (ii) Baldrige healthcare criteria for performance excellence [9]; and (iii) a questionnaire to measure organizational attributes of primary care practices [25]. We did not find any evidence that the instruments designed for use in LMICs [26–29] had been validated. had been validated. Of the tools reviewed, we found the Management Practices Measurement tool most relevant to build on, to measure management practices of health facilities under PBF in Nigeria. It has similar components to the management areas identified by Mabuchi et al. [5] including, performance management, staff management and motivation, and community engagement. Also, it uses an external assessment by trained personnel based on a specific scoring grid and criteria, which addresses capacity constraints and self-reporting bias at PHCCs.

Of the about 40 publications and reports reviewed, 13 intended to define health facility managers’ practices. Table 1 synthesizes the findings from these 13 papers on management practices in health facilities. It identifies seven different management practice areas from practices requiring hard skills (such as financial management) to practices drawing upon much softer skills (such as communication and team building). In addition, the review of elements of health facility autonomy under PBF developed by Fritche et al. [19] and NPHCDA [30] highlighted one further area – pharmaceutical management. Based on these findings, we described 8 key management practice areas for the scorecard, including problem solving, communication, staff and team management, planning, performance management, relationship building and resource mobilization (which was redefined as community/client engagement), financial management and pharmaceutical management. We operationalized the 8 key management areas using relevant indicators from the original Management Practices Measurement tool identified through the literature review. We did not consider indicators that focused on hospital management because these were not relevant for PHCCs in LMICs. Based on findings from the qualitative case studies previously conducted [5], we subsumed communication under the staff management practice area and decided to drop the problem-solving practice as it overlaps with other areas such as planning and target setting and performance management.

#### Step 2: Development of scorecard

Table 2 provides the areas and indicators of the developed management scorecard (Full scorecard available in Appendix A). The scorecard included 32 indicators grouped into 6 broad management practice areas: (i) community/client engagement; (ii) stakeholder engagement; (iii) staff management; (iv) planning and target setting; (v) performance management; and (vi) use of funds and financial management. Each area was broken down into sub-areas (e.g. ‘performance tracking’ and ‘performance review’ for the performance management area) and indicators (e.g. ‘visualization of performance data’ and ‘staff attention to performance’ for the performance tracking sub-area). Ordinal responses derived for each indicator were assigned value of 1, 2 and 3 respectively, resulting of aggregate score range of 32–96 from 32 indicators for each PHCC.

### Statistical analysis

#### Mean and distribution of scores for each scorecard indicator

Complete responses for all indicators on the scorecard were obtained from 111 PHCCs, including 222 respondents (111 OICs and 111 non-OIC workers). Of the 111 PHCCs initially selected, two PHCCs in Ondo state refused the interview because they had just started PBF, and six PHCCs in Adamawa were not accessible due to an insurgency. They were replaced by other PHCCs through random selection. As shown in Table 2, mean scores of the 32 indicators ranged 1.5–2.77, with average 2.16. The standard deviation of the scores ranged 0.33–0.83, with average 0.6.

#### Validity and reliability of the scorecard

Experts review suggested high face validity of the scorecard. Table 3 presents the result of the EFA with PROMAX rotation. Five factors had eigenvalues more than 1, and screeplot shows flattened line at the Factor 7. Given that the commonality was higher for the six-factor model than for the five-factor model, and that our qualitative and literature review described above suggested a six-factor model, we chose the six-factor model dropping 12 indicators with loadings less than 0.4. Since all items except for S5, S22, S31, and S32 had uniqueness higher than 0.50, we kept the items with uniqueness higher than 0.50 as long as their factor loadings were 0.40 or above. Also, three cross-loading items with values ≥ 0.32 on at least two factors were dropped (S9, S17, S20) from this model. As a result, 17 indicators were kept for analysis.

Table 4 summarizes the analysis of the factors presented in the EFA and indicators. The EFA result consists of six factors and 17 indicators. The six factors were named based on the discussion among the authors on grouped items under each factor: – A: Stakeholder engagement and communication; B: Community-level activities; C: Update of plan and target; D: Performance Management; E: Staff attention to planning, target, and performance; and F: Drugs and financial management. The reasoning by the authors behind the names of the six factors is summarized in Table 4. For the ICC, the correlation among mean ratings for each team of judges is 0.94, showing high inter-rater reliability.

## Discussion

We developed a novel scorecard that measures management practices in PHCCs in Nigeria. We highlighted financial management, community and stakeholder engagement as key additional elements of management practices for PHCCs in LMICs in addition to the Management Practices Measurement tool developed by Dorgan et al. [6], Bloom et al. [7], and McConnell et al. [8] for use in high income countries. Our scorecard also introduced a more specific definition of scoring criteria than the original instrument, and questions for non-OIC health workers to enable local data collectors to rate practices and to reduce social desirability bias. These are new and original features of the scorecard that would facilitate its adaptation to capacity-constrained contexts in LMICs.

### Original scorecard vs. EFA results

The developed scorecard was further refined through the EFA. The EFA reduced the number of items from 32 to 17. It also provided a different grouping of items from the originally proposed management practices scorecard based on our qualitative study [5] and literature review. Table 5 compares the originally proposed management practices scorecard with findings based on the EFA results. There are a few notable differences. First, although community engagement is to some extent covered by the latent factor A and B in Table 3, a set of items related to building the relationship with and attracting patients (S1-S4) were not included in the EFA results (see Table 5, right-hand column ‘New Groupings’). These dropped items were however highlighted as key differentiating factors of PHCC performance under the related qualitative study [5]. This may suggest that there are slight differences between factors that relate to PHCC performance and factors that represent health center management (suggested through EFA). Hence, the factors that explain health center management on the one hand and health center performance on the other, may be overlapping but not identical. For example, drugs and financial management are not a factor that directly differentiated high and low performers in the qualitative case study [5], whereas this is an important element of health center management based on the EFA. Likewise, outreach, household visits, and strategies to attract patients may not be a direct element of health center management, though they are key specific approaches that influence the performance of the PHCC. It is noted that community/client engagement is not included in the management practices measurement tool by Dorgan et al. [6], Bloom et al. [7], and McConnell et al. [8], and synthesized key elements of critical primary health facility management (Table 1).

**Table 5.** Re-description of the originally proposed management practices scorecard based on EFA results.

Areas	Sub-Areas	ID	Item	New Groupings
1. Community/Client Engagement	1.1. Community outreach	S1	Outreach	Dropped
		S2	Household visit	Dropped
	1.2. Community trust and satisfaction	S3	Listening and responding to client feedback	Dropped
	1.3. Client recruitment/retention	S4	Patient recruitment and retention activities	Dropped
2. Stakeholder Engagement	2.1. Engagement with Community Leaders	S5	Meetings with community leaders	A. Stakeholder engagement
		S6	Request to community leaders	
		S7	Activities to encourage support from community leaders	B. Community incentive/trust
	2.2. Supervisor engagement	S8	Meetings with supervisors	A. Stakeholder engagement
		S9	Request to supervisors	Dropped
3. Staff management	3.1. Management of staff and working environment	S10	Staff involvement in bonus decision	Dropped
		S11	Team work building	Dropped
		S12	Efforts to improve staff working environment	Dropped
	3.2. Staff communication	S13	Feedback to OIC	Dropped
		S14	Responses from OIC to feedback	A. Stakeholder engagement
		S15	Open communication	Dropped
	3.3. Recognition, Rewarding and punishment of staff	S16	PBF bonus allocation	Dropped
		S17	Rewarding of high-performing staff	Dropped
		S18	Addressing low-performing staff	D. Performance management
4. Planning and Target Setting	4.1. Planning	S19	Business plan update	C. Update of plan and target
		S20	Business plan content	Dropped
		S21	Staff attention to business plan	E. Staff attention to plan, target, and performance
	4.2. Target setting	S22	Target update	C. Update of plan and target
		S23	Setting stretch/achievable targets	D. Performance management
		S24	Staff attention to targets	E. Staff attention to plan, target, and performance
5. Performance Management	5.1. Performance tracking	S25	Visualization of performance data	Dropped
		S26	Staff attention to performance	E. Staff attention to plan, target, and performance
	5.2. Performance review	S27	Regular performance review meeting	Dropped
		S28	Performance review discussions	D. Performance management
6. Use of funds and financial management	6.1. Use of funds	S29	Drug management	F. Drugs and financial management
		S30	Use of PBF funds to attract patient and build trust	B. Community incentive/trust
	6.2. Financial Management	S31	Financial record update	F. Drugs and financial management
		S32	Financial record content	

Another possibility is that the indicators for community/client engagement did not measure the practices sufficiently well. These indicators were developed specifically for this scorecard and could have been flawed. For example, the frequency of outreach last week (S1) may be too short a time period to get a reliable picture of outreach, or this measure may put too much emphasis on frequency and not enough on the quality of outreach. Further formative research, elaboration and testing of the scorecard questions may be needed in this area.

Second, most of the items related to Staff Management in the original scorecard were dropped, and the items kept were assigned to separate groups (i.e. ‘A. Stakeholder engagement’ and ‘D. Performance management’). This is not consistent with the synthesized key elements of critical primary health facility management (Table 1) where activities to assign appropriate roles and responsibilities, create opportunities for learning, motivate and coach health workers, and promote cohesion and teamwork were highlighted as a key element of health facility management. The Management Practices Measurement tool also has ‘Talent management’ in the instrument [6–8]. This may suggest the challenge of scoring such activities in the scorecard, and points to the need for further review and adaptation. At least, however, some dimensions of staff management, such as responsiveness to staff feedback as a part of broader stakeholder engagement, and handling of poor performing staff as a part of performance management are covered in the final factors.

### EFA results vs. literature

The EFA results are consistent with the developed management practices scorecard and literature in other settings. Latent factors ‘C. Update of plan and target’, and ‘D. Performance management’ and the items grouped in these factors are consistent with the Management Practices Measurement tool. Also, the factor ‘F. Drugs and financial management’ is consistent with the synthesized key elements of critical primary health facility management (Table 1), as well as the key management practices for the health facilities to manage the PBF scheme [19]. The latent factor ‘E. Staff attention to plan, target, and performance’ is a different grouping from the original management practices scorecard. However, this demonstrates the importance of communication, involvement, and incentives to motivate staff to be attentive to plan, target and performance, which is consistent with findings in the qualitative case study [5] and the Communication element of the synthesized key elements of critical primary health facility management (Table 1).

### Value and use of the research

This research added significantly to the literature on health center management in developing countries. A careful review of prior studies and application of existing instruments with adjustments, expert review of the scorecard, and high inter-rater reliability are signals of the validity and reliability of the developed measurement approach. The EFA also provided a refined management practices scorecard, despite some differences between the results that it offered and findings from the literature and the related qualitative case study [5].

Capacity building of health facilities is included in most primary health care interventions in developing countries. However, there has been no instrument to help assess management practices and provide critical feedback to improve health facility management to-date. Recent systematic reviews of researches on primary health care systems in LMICs suggest that major research gaps exist in how to improve facility management [31], and that routinely used performance measurement and management strategies are implemented without sufficient knowledge of their effects [32]. This scorecard can help address these critical gaps thus strengthening primary care services. The resulting scorecard is relatively simple, encompassing just 17 different indicators, and includes clear scoring criteria, meaning that it would be relatively straightforward for the central and local government officials to apply the scorecard as part of routine supervisory visits, and not just as part of a research project. This scorecard was used in Nasarawa state of Nigeria to measure baseline and follow-up management scores of the PHCCs under PBF funded by the World Bank to design/guide and measure the result of management strengthening interventions. This indicates high acceptability of the scorecard. Wider application of this scorecard would in turn help to further strengthen the scorecard and guidance associated with it.

### Limitations and areas for further study

As suggested above, one of the limitations to this research and the scorecard is that some of the scorecard questions and scoring criteria, notably those related to community/client engagement, and staff management would benefit from further investigation and refinement. Given the limited literature seeking to assess management practices quantitatively, we were unable to compare our findings to other studies from LMICs.

The scorecard was designed to serve the needs of primary health care facilities under PBF or similar schemes that provide autonomy and funds for the health facilities to improve health services. and it was designed for use in the Nigerian context, drawing in particular on a qualitative case study previously conducted in Nigeria [5]. In order to understand how this scorecard may apply in other contexts, both with and without PBF, further studies may be required using confirmatory factor analysis (CFA) to assess the model fit of the scorecard. Adaptations would also be necessary to assess management practices in settings where there are more limited management autonomy and discretionary funds. Differences in health system structure and function, for example the structure of drug supply systems, or the extent of decentralization, may also influence items and constructs to be included in the scorecard.

## Conclusion

While the management scorecard presented here is undoubtedly an initial attempt to develop a measurement tool that can be used across primary health care settings in low resource environments, we believe that further investment in this objective is warranted. The review by Rowe et al. [2] suggests that management approaches consistently had moderate to large effects on health worker performance. It is time to dismantle and investigate the black box to better understand facility management, how it affects performance, and how it may be strengthened. Ideally management scorecards would be used on a repeated basis, so that primary health care managers as well as central and local government policymakers can see how performance improves over time. Such repeated use may warrant reconfiguration of the scorecard at different time points to respond to the dynamic changes in management practices impacting performance over time. Learning from the related literature on balanced scorecards (e.g. Peters et al. [33], Khan et al. [34]) may be relevant in this regard.

## Paper context

In low/middle-income countries, there is no validated and reliable measurement tool to measure health facility management, and many interventions to improve primary health care (PHC) facilities are designed without an evidence base on what management practices are critical. This paper developed and validated a scorecard to measure management practices at PHC facilities in LMICs. The scorecard is applicable for routine supervisory visits to PHC facilities and can help accumulate knowledge on facility management.

## Appendix Appendix A. Management Practices Scorecard for the PHCCs under PBF in Nigeria

**Table:**

Areas	Sub-Areas	Description	Questions	Examples of Acceptable strategies/activities/ incentives for scoring (Not exhaustive)	Indicator/Scoring Criteria			Q-ID
					1	2	3	
1. Community/ Client Engagement	1.1. Community outreach	The PHCC frequently conducts outreach and household visits	**Outreach** How often did you and your staff conduct outreach last week?		Zero	1-2 times	3 times or more	1
			**Household visit** Do you and your staff always visit individual households during the outreach? (To verify - Who do you visit? What do you do at each household?)		PHCC do not carry out household visits, or they failed to explain who they target and what they do in each household	PHCC sometimes visits households and/or is vague about who they target and what they do in each household.	Always - PHCC could explain who they target (e.g., pregnant women, under-five children) and what they do in each household.	2
	1.2. Community trust and satisfaction	The PHCC builds trust and improves satisfactions of the community members	**Listening and responding to client feedback** - Does the PHCC have a functional suggestion box, or other means to collect complaints?- In recent 6 months, have you made any changes based on a suggestion from patients? Please explain.		PHCC did not have a functional suggestion box or other specific means to collect client feedback.	PHCC has a suggestion box or other means, but have not made changes based on the client feedback in recent 6 months.	PHCC has a suggestion box or other means, and had made changes based on the feedback in recent 6 months.	3
	1.3. Client recruitment and retention	The PHCC carries out multiple forms of strategies to attract and retain patients	**Patient recruitment and retention activities**What have you done to attract patients to come to and continue to use PHCCs?	**1. Standard approach**- Outreach to explain value of PHCC- Communications through community leaders,- Physical incentives such as gift, **2. Advanced strategy (1) Communication and partnership**- Individual follow-up with pregnant women, - Partnership with TBAs and other providers **(2) Incentives**- Public recognition of those attending, - Free transport service, (3) **Service improvement**- Improvement of maternity ward,- TV and other services to improve experiences at PHCCs- Friendly/respectful communications (with distinctive examples),	PHCC does not carry out standard approach comprehensively.	PHCC carries out standard approach but use of advanced strategy is limited to 1 or 2 advanced strategies mentioned.	PHCC carries out standard approach and 3 or more advanced strategies in the criteria to attract patients	4
2. Stakeholder Engagement	2.1. Engagement with Community Leaders	The PHCC involves community leaders (e.g., community leaders, traditional/religious leaders, youth leaders) in important decision making and problem solving, and gains support from them. The PHCC places clear requests to WDC and community leaders, and Incentivize their support.	**Meetings with community leaders** In the last 30 days, how many times did you or your staff meet or speak with Ward Development Committee (WDC) and community leaders?		Zero or once.	2-3 times.	4 times or more.	5
			**Request to community leaders** In the last 30 days, have your PHCC asked specific support from WDC or community leaders to improve quantity or quality of PHCC services? What is it?	- Address/regulate quacks, - Educate, inform, and encourage community to visit PHCCs, - Facilitate labor contribution by community,- Identify those who do not use PHCCs and address them,- Advise decision on the use of PBF funds	- PHCC did not ask any specific support to WDC and/or community leaders in the recent month.	- PHCC asked 1 or more support in the recent month, but requests are not specific for their actions.	- PHCC asked 1 or more specific support in the recent month.	6
			**Activities to encourage support from community leaders** What does your PHCC do to encourage or appreciate the support from WDC and community leaders? Probe if needed:- Do you provide any gift or financial incentive to them?- Do you meet with them to report the performance of your PHCC? - Do you publicly recognize their support? How?	- Cash, gifts, - Free or discounted service to family, - Public recognition of their contribution, - Involvement in decision making, - Frequent meeting and reporting	No specific rewards or incentives are provided to WDC and community leaders.	1-3 specific rewards or incentives in the criteria are provided to WDC and community leaders.	4-5 forms of incentives in the criteria are explained.	7
	2.2. Supervisor engagement	PHCC frequently consults with LGA, SPHCCDA supervisors, or consultants on utilization and quality issues, receive various helps.	**Meetings with supervisors** In the last 30 days, how many times did you or your staff meet or speak with LGA or SPHCCDA supervisors, or other supervisors (e.g., TA consultants)?		Zero or once.	2-3 times.	4 times or more.	8
			**Request to supervisors** In the last 30 days, have your PHCC asked specific support from LGA or SPHCCDA supervisors to improve quantity or quality of PHCC services? What is it?	- Technical advice on the use of PBF bonus or other issues,- Technical advice on specific indicators to improve results, - Engage with community leaders to educate community and/ or address quacks,	- PHCC did not ask any specific support to supervisors in the recent month.	- PHCC asked 1 or more support in the recent month, but requests are not specific for their actions.	- PHCC asked 1 or more specific support in the recent month.	9
3. Staff management	3.1. Manage-ment of staff and working environment	The PHCC manages staff and creates an environment in a way that it enhances staff motivation and teamwork and ensures employee safety and satisfaction.	**Staff involvement** For the last PBF bonus received, who decided the way to use the bonus? (Probe: Are there staff who were not involved in that decision?)		PHCC staff are not involved in the decision on the use of PBF bonuses and other revenue.	Some but not all of PHCC staff are involved in the decision on the use of PBF bonuses and other revenue.	All PHCC staff are always involved in the decision on the use of PBF bonuses and other revenue for improving health facilities.	10
			**Team work building** What do you do to build team work and encourage them to help each other?	- Covering up staff's absence, - Team events, - Providing food to eat together, - Discuss team work in staff meeting, - Proactively resolve or set-up a system to resolve staff conflicts	No clear activities are mentioned for building team work and collaboration.	1 or 2 specific activities are mentioned for building team work and collaboration.	Combination of multiple efforts are made to enhance team work.	11
			**Efforts to improve staff working environment** How do you improve staff's working environment and facilitate their outreach activities?	- Staff housing, - Security, - Finance transport for outreach, - Hire extra staff to address staff shortage, - Create staff training opportunities, - Make sure to prevent drugs and supplies stock out	No clear activity in the criteria was explained for staff's working environment and their outreach activities.	1-2 activities in the criteria for staff's working environment and their outreach activities are mentioned.	3 or more activities in the criteria for staff's working environment and their outreach activities are mentioned.	12
	3.2. Staff communica-tion	PHCC creates an atmosphere and process for open communication between staff, and staff and OIC	**Feedback to OIC** (to non-OIC health worker) (1) When is the last time you provided your suggestions to the OIC to improve use and quality of PHCC?		Staff did not provide any opinion in the last one month.	Less than a month ago.	Less than a week ago.	13
			**Responses from OIC to feedback** (to non-OIC health worker) Is OIC receptive to your opinion, and act on your suggestions?		No	To some extent	Always	14
			**Open communication** (to non-OIC health worker) Does OIC make visible efforts to encourage open communication?		No	To some extent	Always	15
	3.3. Recognition, Rewarding and punishment of staff	Staff's good practices and high performance are recognized and rewarded	**PBF bonus allocation** (to non-OIC health worker) How performance bonuses are allocated? Do staff at this facility see the allocation of the performance bonus as fair?		Unfair	Neutral	Fair	16
		Staff's good practices and high performance are recognized and rewarded, while poor performance and bad behavior are addressed appropriately.	**Rewarding of high-performing staff** In addition to bonus allocation, how are the staff rewarded based on performance? (Probe: public recognition/award, additional cash or gifts, training opportunities, more responsibility/promotion?)	- Public recognition/award, - Additional cash or gifts, - Training opportunities, - More responsibility/promotion	No other rewards than the performance bonus allocation are provided.	In addition to the performance bonus allocation, one more form or reward is provided.	2 or more forms of rewards are provided.	17
			**Addressing low-performing staff** How are those who perform poorly addressed? Probe:- Have anyone been fired or replaced because of poor performance?- Have the bonus to them been reduced and reasons given?- Are they monitored closely by OIC, based on improvement targets?	**Minimum activities**- Low performance bonus, - Individual and public feedback, **Advanced activities**- Agreement on improvement plan and follow-up on implementation, - Monetary penalties,- Termination of employment or transfer,- staff change	No actions are made to poor performers.	Minimum activities in the criteria are made to poor performers.	Minimum activities and some of the advanced activities in the criteria are made to poor performers.	18
4. Planning and Target Setting	4.1. Planning	The PHCC has robust business plan with targets, analysis of problems, and actions to resolve them and achieve targets. The plan is updated regularly, and staff know and feel ownership to the plan.	**Business plan update** When is the last time the business plan was updated?		There is no update on the business plan since it was developed, or it has not been updated more than a year.	The business plan was updated within 12 months.	The business plan is updated within last 3 months.	19
			**Business plan content** (**Observation** of the business plan) Does the plan describe targets, problems in achieving the targets, key activities to address them, timeline, and budget for them?		The business plan includes none of them.	The business plan includes some of them.	The business plan includes all of them.	20
			**Staff attention to business plan** (to non-OIC health worker) What are the key issues and activities explained in the business plan?		The staff do not know about the business plan.	The staff have some knowledge about the activities in the business plan (e.g., priority activities)	The staff is fully aware of the content of the business plan (at least target, issues, and key activities).	21
			**Target update** When is the last time the targets were updated?		There is no update on the targets since they were set, or it has not been updated more than a year.	The targets were updated within last 12 months.	The targets were updated within last 3 months.	22
	4.2. Target setting	Targets on the utilization of key services and quality score for key areas are up-to-date, specific, demanding and achievable, and known by staff.	**Target stretch/ achievable** (For an example of “institutional delivery” indicator - ask only if the target was updated within last 12 months) Why is the current target updated? (Prove: how does it relate to current performance? How is it achievable?)	**Scoring criteria**: The updated target should be: (i) based on the current performance; (ii) demanding; and (iii) achievable.	The target is not updated (Score 1 in the previous question), or does not meet any of the criteria	The targets meet some of the criteria.	The targets meet all of the criteria.	23
			**Staff attention to targets** (to non-OIC health worker) What are the key indicators and targets of the PHCC?		The staff do not know about the targets.	The staff have some knowledge about the key indicators and targets (e.g., can tell what are key indicators for the PHCCs).	The staff is fully aware of the key indicators and targets (e.g., key target numbers and actuals).	24
5. Performance Management	5.1. Performance tracking	PHCCs track their quantity and quality performance regularly in a visible way. Performance is communicated to staff and staff are aware of the situation	**Visualization of performance data**(Question and **Observation**) How are the quantity and quality performance data recorded and presented in the PHCC? (Verify physically)		Quantity and quality of services are not recorded regularly (monthly).	Quantity and quality of services are recorded regularly (monthly) but not visualized so that all staff and patients can always see them.	Quantity and quality of services are recorded regularly (monthly) and visualized (e.g., on a wall as a graph or table) so that all staff and patients can always see them.	25
			**Staff attention to performance** (to non-OIC health worker) What are the performance of last month for key indicators (e.g., institutional delivery, OPD, quality score) of the PHCC?		The staff do not know about the actual performance.	The staff can explain the actual performance for at least one key indicator.	Staff is fully aware of the actual performance for key indicators (e.g., institutional delivery, OPD, quality score).	26
	5.2. Performance review	PHCC carries out regular performance review meetings with key stakeholders and develops activities based on the review results.	**Regular performance review meeting** Are there regular meeting at the PHCC to review performance, with staff, WDC and community leaders?		There is no regular performance review meetings at the PHCC.	The performance review meeting is held at the PHCC every month or quarter. However, WDC and community leader, and other stakeholders do not participate or their participation is not regular.	The performance review meeting is held at the PHCC every month or quarter, and staff, WDC, and community leaders participate in the meeting.	27
			**Performance review discussions** To review the contents of the meeting: - What was discussed in the last meeting? (Probe: performance? agreed actions to improve performance?)		There is no discussion on performance, and actions to improve performance.	The OIC explained clearly how performance was reviewed, but agreed actions to improve performance were not very specific.	The OIC explained clearly how performance was reviewed, and agreed actions to improve performance were very specific.	28
6. Use of funds and financial management	6.1. Use of funds	PHCC uses performance bonus and other funds received to the area that directly improve patient use and quality of services.	**Drug management** In the last quarter, had the PHCC experienced any stock-outs? (To verify, **observe** the storage room whether the room is fully equipped with drugs)		PHCC experienced stock-outs more than 3 times n the last quarter.	PHCC experienced stock-outs 1-2 times in the last quarter.	None. Storage room has no stock-outs.	29
			**Use of PBF funds** In addition to the funds for drugs and supplies: - In the recent three months, what did the PHCC use PBF bonus or other funds for? - Why did the PHCC use funds for these activities?	**Patient attraction**: - Incentives/gifts to pregnant women for visit to PHCCs; fee reduction, fee waiver, transport support, etc.; **Patient trust**: - Hiring of additional staff (e.g., medical doctors, staff from community. staff to enable 24H service); - Improvement of maternity ward or other services that attract patients;**Outreach**: - Transport for outreach**Quality of care**: - Any areas in the quality checklist that the PHCC does not achieve/score	PHCC does not invest in the areas that directly help attract patients, build patient trust, strengthen outreach activities, or improve quality of care.	PHCC use funds for a few activities that directly help attract patients, build patient trust, strengthen outreach activities, or improve quality of care.	PHCC invests in the area that directly help attract patients, build patient trust, strengthen outreach activities, and improve quality of care in multiple ways.	30
	6.2. Financial Manage-ment	PHCC records incomes and expenses clearly, reviews the financial records regularly, and plans the activities based on available funds.	**Financial record update** (**Observation**) How often do your PHCC update the financial book? (To verify, review the actual financial book)		The financial records are not updated.	The financial records are updated with some delays, and monthly balance is not available.	The financial records are updated each time there is expense, and revenue and expenses are balanced every month.	31
			**Financial record content** (**Observation**) Can we see the financial record of the PHCC? - Check whether incomes and expenses for the last month are recorded clearly, and total balance of the month or quarter is calculated correctly.		The incomes and expenses are recorded poorly or not recorded, and the calculation does not match.	The incomes and expenses are recorded OK.	The incomes and expenses, and the total balance of the month or quarter are calculated correctly.	32
